# Impact of short-term online continuing education on gastroesophageal reflux disease on the cognitive level and diagnostic and therapeutic behaviors of physicians in medical consortiums

**DOI:** 10.3389/fpubh.2026.1821119

**Published:** 2026-05-28

**Authors:** Yibing Zhang, Jie Jin, Yuping Yuan, Yifan Ke, Junyan Du

**Affiliations:** Department of Gastroenterology, Wenzhou Central Hospital (The Dingli Clinical College of Wenzhou Medical University), Wenzhou, Zhejiang Province, China

**Keywords:** cognitive level, diagnostic and therapeutic behaviors, gastroesophageal reflux disease, medical consortiums, online continuing education

## Abstract

**Objective:**

To explore the impact of short-term online continuing education on gastroesophageal reflux disease (GERD) on the cognitive level, diagnostic and therapeutic behaviors of physicians in medical consortiums.

**Methods:**

Physicians of different levels, titles, and specialties from member units of the medical consortium were invited to receive an online GERD training program via the DingTalk platform and complete knowledge tests. The differences in knowledge test scores before and after training were analyzed, and the differences in physicians' choices of GERD examination methods and treatment plans before and after training were evaluated.

**Results:**

A total of 232 physicians completed the study. The total score of the knowledge test after training was significantly higher than that before training (*P* < 0.05). The increase in knowledge test scores of physicians from primary and secondary hospitals, general practitioners and internal medicine physicians were significantly higher (*P* < 0.05). Data analysis using the GEE model showed that compared with before training, physicians were more likely to choose gastroscopy and gastrointestinal motility tests after training (*P* < 0.05). Gastroenterologists were more likely to choose gastrointestinal motility tests than general practitioners, and physicians from tertiary hospitals were more likely to choose gastrointestinal motility tests than those from primary hospitals (*P* < 0.05). Compared with before training, physicians were more likely to choose lifestyle modifications after training (*P* < 0.05).

**Conclusion:**

Short-term online continuing education on GERD can effectively improve the cognitive level of physicians in medical consortiums, especially for physicians in primary hospitals and general practitioners.

## 1 Introduction

Gastroesophageal reflux disease (GERD) is a common clinical gastrointestinal disease. The prevalence of GERD in China is approximately 1.9%−7.0% (1), showing an increasing trend year by year. GERD is a condition that consumes substantial medical resources and has become one of the major digestive diseases seriously affecting people's quality of life (2). Currently, the diagnostic methods for GERD mainly include symptom questionnaires, gastroscopy, and gastrointestinal motility tests (24 h esophageal acidimetry and esophageal manometry). Treatment measures include lifestyle modifications, medications (such as proton pump inhibitors), and surgical treatment. Physicians in medical consortiums, especially those in primary hospitals, still have a relatively backward understanding of GERD, and their diagnosis and treatment of GERD are not standardized, failing to achieve the expected efficacy. It is crucial to standardize the diagnosis and treatment of GERD among all physicians in medical consortiums. At present, with the development and popularization of the Internet and smartphones, online continuing medical education courses have two major advantages: convenience and affordability (3). Current domestic and foreign studies on online continuing medical education mostly focus on the improvement of theoretical knowledge, with little involvement in changes in clinical practice behaviors. Therefore, we conducted a study using the consensus opinions on GERD as educational content and the DingTalk software as the online learning platform to explore the impact of short-term online GERD continuing education on the cognitive level, especially the diagnostic and therapeutic behaviors, of physicians in medical consortiums. The results are reported as follows.

## 2 Materials and methods

### 2.1 General information

From January 2022 to June 2023, a total of 252 clinicians from different levels of hospitals, with different professional titles and specialties in member units of the medical consortium were included to participate in the short-term online continuing education program on GERD. Inclusion criteria: ① Obtained a practicing physician qualification certificate of the People's Republic of China; ② Clinical physicians with a daily outpatient volume of more than 15 person-times; ③ Specialties: gastroenterology, internal medicine, or general practice; ④ Voluntarily participated in this study and signed an informed consent form. Exclusion criteria: ① Those who were not proficient in operating the DingTalk platform or unable to operate it; ② Those who could not provide GERD case information or had fewer than 3 cases. This study conforms to the medical research ethics principles in the Declaration of Helsinki.

### 2.2 Methods

#### 2.2.1 Online continuing education

The continuing education platform used in this study was the DingTalk APP. A GERD training program for physicians in the medical consortium was created on DingTalk, with an online classroom opened to release multiple training videos, such as the 2020 Chinese Expert Consensus on Gastroesophageal Reflux Disease (4), the 2020 Chinese Expert Consensus on Endoscopic Treatment of Gastroesophageal Reflux Disease, and the Chinese Multidisciplinary Diagnosis and Treatment Consensus on Gastroesophageal Reflux Disease, as well as GERD knowledge test questions. The training program consisted of 3 core modules with a total of 3 hours of video content: Module 1 (60 min): 2020 Chinese Expert Consensus on GERD (including diagnostic criteria, classification and surgical indications); Module 2 (60 min): Interpretation of functional diagnostic tests (24 h esophageal pH monitoring and esophageal manometry); Module 3 (60 min): Standardized treatment of GERD (lifestyle modifications, pharmacotherapy and endoscopic treatment). All modules included pre-recorded lectures, case demonstrations and interactive quizzes. The learning period was 1 month, and physicians could watch the videos repeatedly at their convenience. All knowledge tests and clinical case submissions were completed within 1 week after the end of the training period.

Module 1 also included detailed explanations of predefined surgical treatment indications according to the 2020 Chinese Expert Consensus: ① Refractory GERD (no symptom improvement after 8 weeks of standard PPI treatment); ② PPI dependence (need for long-term PPI maintenance to control symptoms); ③ Complicated with large hiatal hernia (≥2 cm); ④ Presence of GERD complications (Barrett's esophagus with dysplasia, recurrent esophageal stricture); ⑤ Patient preference for surgical treatment after adequate informed consent.

Module 2 (60 min): Interpretation of functional diagnostic tests, including: ① Normal reference values and abnormal judgment criteria for 24 h esophageal pH monitoring (DeMeester score, total acid exposure time, number of reflux episodes); ② Normal reference values and abnormal judgment criteria for esophageal manometry (lower esophageal sphincter pressure, esophageal body peristalsis function); ③ Clinical significance of abnormal results and their impact on treatment decisions.

#### 2.2.2 Knowledge test content and clinical diagnosis and treatment case content

The GERD knowledge test content included 5 parts: (understanding of symptoms, diagnostic criteria, examination methods, treatment plans, and refractory GERD) to assess physicians' cognitive level. The test was conducted once before and once after training. The knowledge test consisted of 25 questions, each worth 4 points, with a total score of 100 points. The clinical diagnosis and treatment cases of GERD mainly included medical history, examination methods, and treatment, and each case required a detailed description of the diagnosis and treatment plan for the GERD patient. All clinical cases were standardized to include typical GERD (reflux esophagitis) and atypical GERD (extraesophageal manifestations) presentations. Physicians were required to submit 1 complete clinical case within 1 week before training and 2 complete clinical cases within 1 week after training. The time windows for training and case submission were identical for all participants to eliminate temporal bias.

### 2.3 Evaluation indicators

The total score of the GERD knowledge test reflected physicians‘ cognitive level of GERD, with a higher score indicating a higher cognitive level. The change in physicians' cognitive level of GERD was reflected by comparing the increase in the total score of the GERD knowledge test before and after training. Through the data of submitted clinical diagnosis and treatment cases of GERD, the differences in physicians‘ choices of examination methods and treatment plans before and after training were determined; further, the changes in physicians' diagnostic and therapeutic behaviors before and after training were reflected through these differences.

### 2.4 Statistical methods

All data in this study were statistically analyzed using SPSS 23.0 software. Measurement data not following a normal distribution were expressed as median (interquartile range), and the comparison between two groups was performed using the paired-sample Wilcoxon test. The GERD case information submitted by each physician was regarded as a group, and there was a correlation between the data within each group. Therefore, generalized estimating equations (GEE) were used for data analysis to evaluate the differences in physicians' choices of examination methods and treatment plans before and after training. A two-tailed test was used, and *P* < 0.05 indicated a statistically significant difference.

## 3 Results

### 3.1 General information

A total of 252 physicians were included in this study, among whom 20 did not complete the knowledge test or had incomplete case information, and 232 actually completed the study. There were 156 males (67.2%) and 76 females (32.8%), aged 25–63 years, with an average age of (38.1 ± 7.5) years.

### 3.2 Comparison of total scores of GERD knowledge test before and after training

As shown in Table 1, the total score of the knowledge test after training was significantly higher than that before training (*z* = −13.21, *P* < 0.05). The increase in knowledge test scores of physicians from primary and secondary hospitals was significantly higher than that of physicians from tertiary hospitals (*H* = −53.78, −76.70, *P* < 0.05, Table 2). Table 3 shows that the increase in knowledge test scores of general practitioners and internal medicine physicians was significantly higher than that of gastroenterologists (*H* = −113.62, −112.71, *P* < 0.05). Subgroup analysis of knowledge test questions showed that the accuracy rate of questions related to surgical treatment indications increased from 41.4% before training to 78.4% after training (*P* < 0.001), and the accuracy rate of questions related to functional test interpretation increased from 35.3% to 72.8% (*P* < 0.001).

**Table 1:** Comparison of knowledge test scores before and after training (points).

Index	After training	Before training
*n*	232	232
Understanding of symptoms	8 (4, 12)	16 (12, 16)
Diagnostic criteria	12 (8, 16)	12 (12, 16)
Examination methods	12 (8, 12)	16 (12, 16)
Treatment plans	20 (16, 24)	20 (20, 24)
Refractory GERD	4 (4, 8)	8 (8, 12)
Total score	52 (44, 72)^*^	72 (64, 80)

^*^, Compared with after training, *P* < 0.05.

**Table 2:** Comparison of knowledge test scores among hospitals of different levels (points).

Group	*n*	Before training	After training	Increase
Primary hospitals	70	44 (40, 56)	64 (60, 76)	20 (20, 24)^*^
Secondary hospitals	54	68 (44, 80)	76 (64, 84)	12 (8, 20)^*^
Tertiary hospitals	108	58 (44, 76)	72 (64, 84)	8 (14, 20)

^*^, Compared with tertiary hospitals, *P* < 0.05.

**Table 3:** Comparison of knowledge test scores among different specialties (points).

Group	*n*	Before training	After training	Increase
General practice	114	44 (40, 52)	64 (60, 72)	20 (20, 24)^*^
Internal medicine	34	44 (40, 50)	64 (60, 72)	20 (12, 20)^*^
Gastroenterology	84	76 (68, 81)	84 (79, 88)	8 (8, 12)

^*^, Compared with gastroenterology, *P* < 0.05.

### 3.3 Analysis of physicians' choices of GERD examination methods and treatment plans

**Figure 1:**
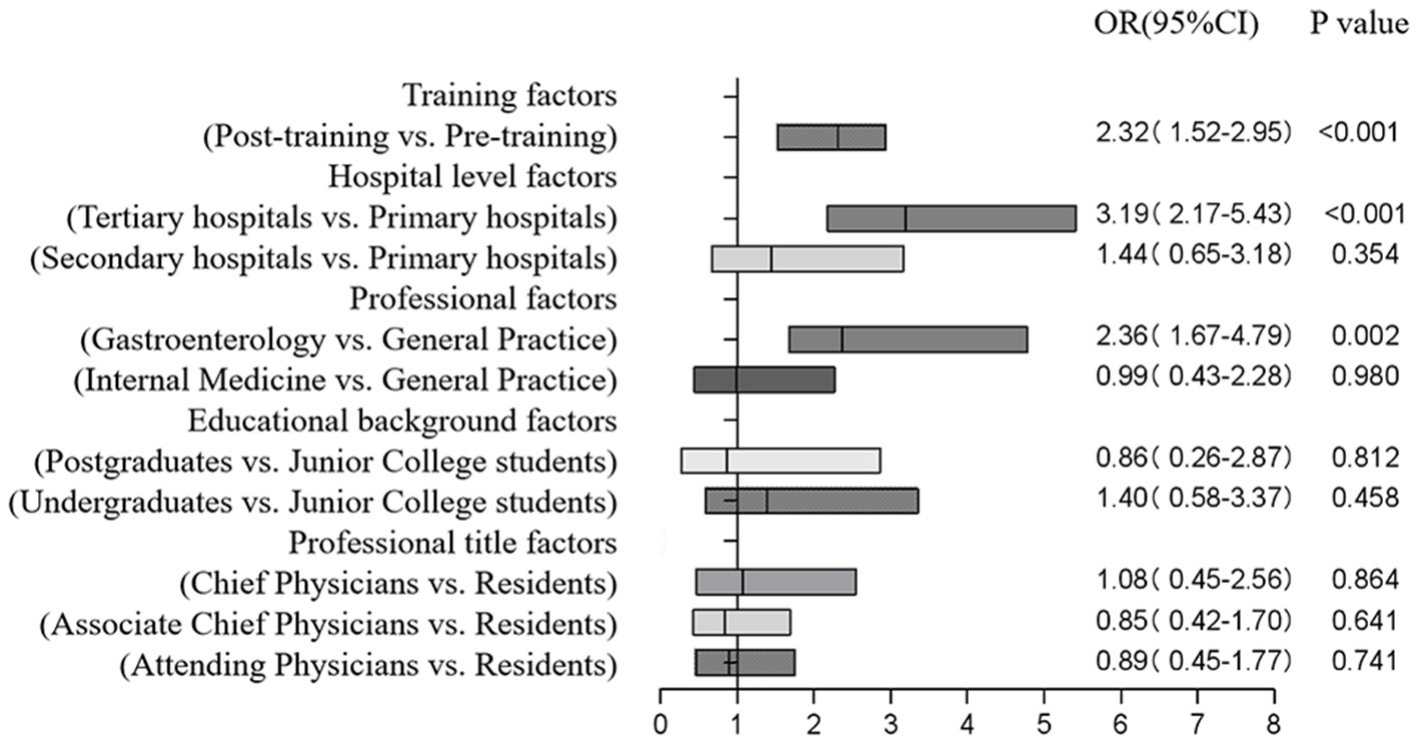
GEE model for physicians' choice of gastroscopy.

**Figure 2:**
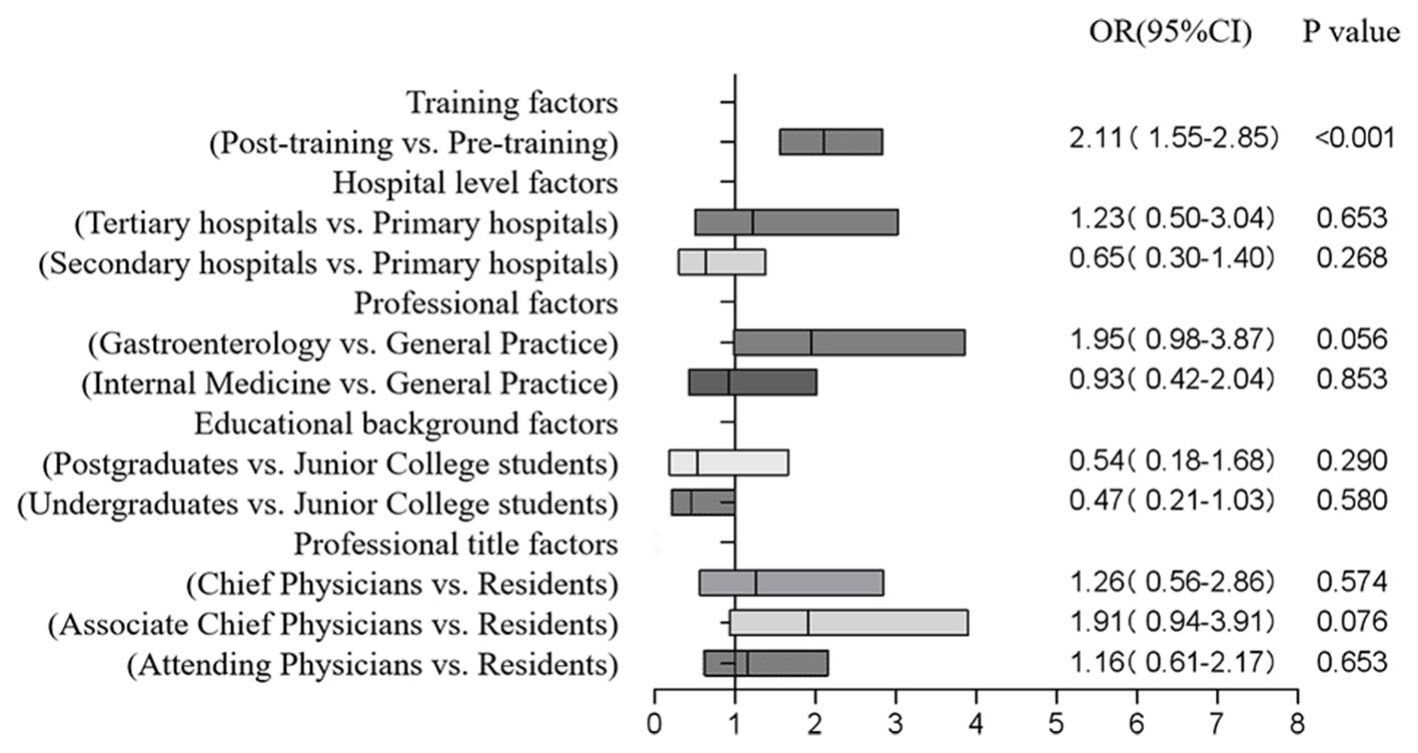
GEE model for physicians' choice of gastrointestinal motility tests.

**Figure 3:**
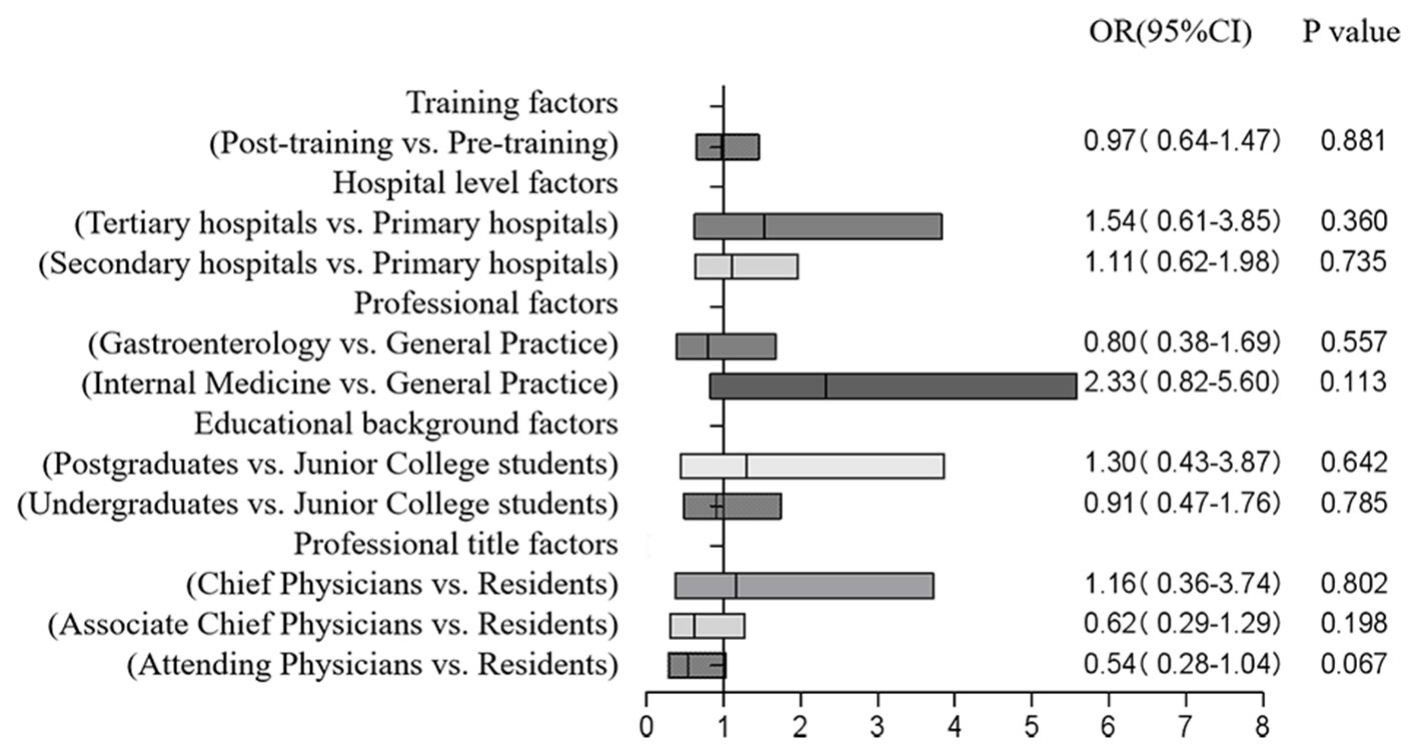
GEE model for physicians' choice of PPI drugs.

**Figure 4:**
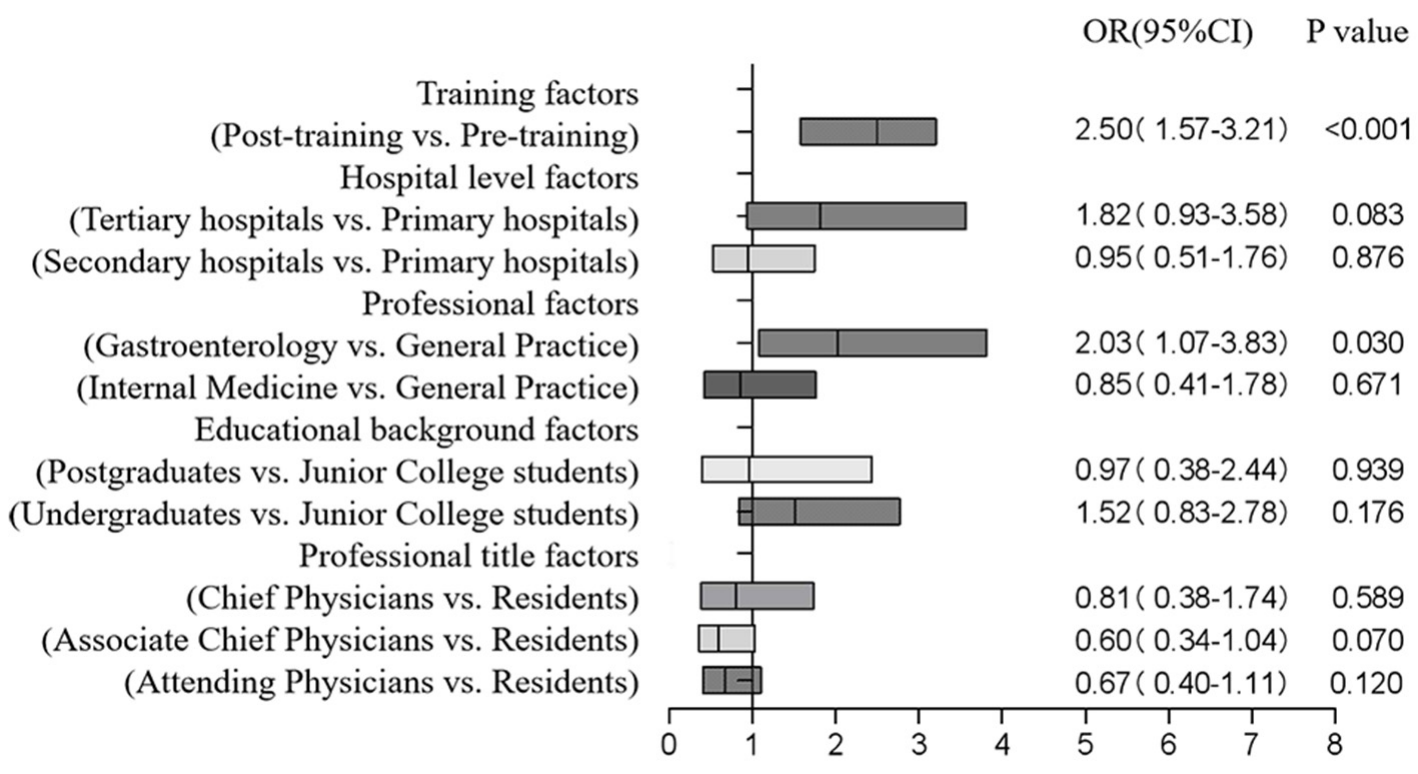
GEE model for physicians' choice of lifestyle modifications.

Data analysis using the GEE model showed that compared with before training, physicians were more likely to choose gastroscopy after training (*OR*=2.11, 95%*CI*=1.55–2.85, *P* < 0.05, Figure 1). As shown in Figure 2, compared with before training, physicians were more likely to choose gastrointestinal motility tests (24 h esophageal acidimetry and esophageal manometry) after training (*OR* = 2.32, 95% *CI* = 1.52–2.95, *P* < 0.05). Gastroenterologists were more likely to choose gastrointestinal motility tests than general practitioners, and physicians from tertiary hospitals were more likely to choose gastrointestinal motility tests than those from primary hospitals (*OR* = 2.36, 3.19, 95% *CI* = 1.67–4.79, 2.17–5.43, *P* < 0.05). There was no statistically significant difference in the selection of proton pump inhibitors by physicians after training compared with before training (*OR* = 0.97, 95% *CI* = 0.64–1.47, *P* > 0.05, Figure 3). Figure 4 shows that physicians were more likely to choose lifestyle modifications after training compared with before training (*OR* = 2.50, 95% *CI* = 1.57–3.21, *P* < 0.05). Further analysis of the 696 submitted clinical cases showed that the consistency rate between physicians' diagnoses and the 2020 Chinese GERD Consensus increased from 62.5% before training to 87.9% after training (χ^2^ = 52.37, *P* < 0.001), and the standardization rate of treatment plans increased from 58.6% to 84.5% (χ^2^ = 57.82, *P* < 0.001).

## 4 Discussion

A medical consortium integrates medical resources within the same region, usually consisting of a tertiary hospital, secondary hospitals (county hospitals), and primary hospitals (community hospitals) in a region. Therefore, the disease diagnosis and treatment capabilities of physicians in medical consortiums are inevitably uneven. Many regions have also explored new training models for physicians in medical consortiums, achieving good results (5–7). Continuing medical education provides physicians with efficient, high-quality, and highly relevant learning courses (8). Online continuing education breaks the limitation of geographical distance, allowing more physicians to participate. It decentralizes and liberalizes time, making it more suitable for busy frontline clinicians to reasonably use fragmented time for self-improvement. Online continuing education can improve physicians‘ satisfaction, self-confidence, and knowledge level (9, 10). During the COVID-19 pandemic, Korean physicians received online medical education, which ultimately affected their clinical capabilities and promoted changes in clinical practice (11). Also during the COVID-19 pandemic, it was reported that Chinese scholars carried out continuing education for endoscopists through online platforms, which was proven to effectively improve endoscopic diagnostic capabilities (12). This study found that the total score of the GERD knowledge test of physicians after training was significantly higher than that before training, which fully indicates that online continuing education can improve physicians' cognitive level of GERD, and the results are similar to those of similar foreign studies (8). It is particularly noteworthy that compared with tertiary hospitals, the improvement in primary and secondary hospitals was more significant; compared with gastroenterology, the improvement in general practice and internal medicine was more significant. This also reflects that physicians in primary hospitals or non-specialized departments of medical consortiums, although having inherent disadvantages before GERD training, can also make up for their shortcomings through online continuing education.

In recent years, some researchers have pointed out that merely increasing professional theoretical knowledge should not be the main goal of continuing medical education. Only when the knowledge acquired by those receiving continuing education can be transformed into changes in medical behaviors in clinical practice can its true educational significance be realized (13). Multiple foreign studies have shown that online continuing medical education can change physicians‘ clinical practice capabilities (11, 14). This study found that compared with before training, physicians in medical consortiums after training had significant changes in the choice of examination methods for diagnosis. After training, they were more active in choosing gastroscopy and gastrointestinal motility tests (24 h esophageal acidimetry and esophageal manometry), especially for physicians in tertiary hospitals and gastroenterology departments, which is also conducive to the standardization of diagnosis. In addition, there was significant progress in treatment, with more active choices of lifestyle adjustments and modifications. However, there was no significant change in the choice of PPI drugs. A possible reason is that PPI drugs have become very common in medical consortiums, and physicians have widely used them in the usual treatment of GERD. In addition, for non-pharmacological therapies, lifestyle modifications are very necessary and have been accepted by physicians after training. This reflects that short-term online continuing education has changed physicians' clinical practice behaviors, providing strong support for the standardized diagnosis and treatment of diseases. Based on the “Internet +” platform, the integration of medical resources has been well realized, promoting the efficient and sustainable development of medical consortiums (15).

This study also has some limitations. First, only immediate post-intervention assessments were conducted, and no long-term follow-up was performed, so it is impossible to determine whether the observed improvements in knowledge and diagnostic/therapeutic behaviors are durable. Future studies will add 3–6 month follow-up assessments to evaluate the long-term impact of online continuing education on physicians‘ clinical practice. Second, it did not involve the clinical efficacy of physicians' diagnosis and treatment cases after training. This will be improved in future studies on functional gastrointestinal diseases, and online continuing medical education will be continuously promoted to make the diagnosis and treatment of functional gastrointestinal diseases more standardized among all physicians in medical consortiums.

## 5 Conclusion

In conclusion, short-term online continuing education on GERD can effectively improve the cognitive level and standardize the diagnostic and therapeutic behaviors of physicians in medical consortiums, which is particularly valuable in settings with limited access to specialized care. However, in borderline or complex cases-especially those requiring interpretation of specialized diagnostic tests or consideration of surgical treatment-the involvement of a gastroenterology specialist remains essential to ensure optimal patient outcomes.

## Data Availability

The original contributions presented in the study are included in the article/supplementary material, further inquiries can be directed to the corresponding author.

## References

[1] Lu TL Li SR Zhang JM Chen CW. Meta-analysis on the epidemiology of gastroesophageal reflux disease in China. World J Gastroenterol. (2022) 28:6410–20. doi: 10.3748/wjg.v28.i45.6410 36533111 PMC9753054

[2] Maret-Ouda J Markar SR Lagergren J. Gastroesophageal reflux disease: a review. JAMA. (2020) 324:2536–47. doi: 10.1001/jama.2020.21360 33351048

[3] Richardson ML Norris TE. On-line delivery of continuing medical education over the World-Wide Web: an on-line needs assessment. Am J Roentgenol. (1997) 168:1161–4. doi: 10.2214/ajr.168.5.9129405 9129405

[4] Chinese Chinese Society of Gastroenterology Chinese Medical Association. 2020 Chinese expert consensus on gastroesophageal reflux disease. Chin J Dig. (2020) 40:649–63.

[5] Lu X Tang H Xu T Song X Jiang F Zheng X . The significance of three-dimensional team management in the medical community model for patients with hypertension and diabetes. J Healthc Eng. (2022) 2022:1960030. doi: 10.1155/2022/1960030 35444777 PMC9015871

[6] Zheng Y Hu J Li L Dai T. Practice and enlightenment of chronic disease management at the county level in china from the perspective of professional integration: a qualitative case study of Youxi County, Fujian Province. Int J Integr Care. (2023) 23:6. doi: 10.5334/ijic.7550 PMC10417912 37577141

[7] Wang Y Huang J Zhang D Jiang J Xue Y Zhao W . Comparing primary-tertiary multidisciplinary collaborative care with usual care in community patients with atrial fibrillation: the protocol for a cluster randomized controlled trial. Contemp Clin Trials. (2025) 154:107957. doi: 10.1016/j.cct.2025.107957 40398599

[8] McMahon GT. Advancing continuing medical education. JAMA. (2015) 314:561–2. doi: 10.1001/jama.2015.7094 26192406

[9] Thepwongsa I Kirby CN Schattner P . Online continuing medical education (CME) for GPs: does it work? A systematic review. Aust Fam Physician. (2014) 43:717–21. 25286431

[10] Ames ML Staffier KL Kees A Freeman K Shetty P Gittelsohn J . Online lifestyle medicine continuing medical education (CME) course completion predicts increases in clinician knowledge, confidence, and practice of lifestyle medicine. Am J Lifestyle Med. (2024) 15598276241279523. doi: 10.1177/15598276241279523 39540184 PMC11556669

[11] Jang A Kim MR Lee SMK Ha IH Shin JY McClain R . Evaluating the effectiveness of online continuing medical education during the COVID-19 pandemic. Med Teach. (2023) 45:852–8. doi: 10.1080/0142159X.2023.2183787 37013818

[12] Li G Yu T Zhang L Du H Zhang W Hou S. Use of a specialty endoscopy online platform for continuing medical education for clinical endoscopists during the COVID-19 pandemic. BMC Med Educ. (2022) 22:458. doi: 10.1186/s12909-022-03516-2 35705967 PMC9198611

[13] VanNieuwenborg L Goossens M De Lepeleire J Schoenmakers B. Continuing medical education for general practitioners: a practice format. Postgrad Med J. (2016) 92:217–22. doi: 10.1136/postgradmedj-2015-133662 26850504 PMC4819632

[14] Shalabi KM Almurdi MM. Satisfaction and attitudes towards online continuous medical education and its impact on clinical practice among physiotherapists. BMC Med Educ. (2024) 24:70. doi: 10.1186/s12909-024-05049-2 38233905 PMC10795308

[15] Qi H Ying Y Zhu L Li Q Wang T Chen B . Exploration on the development of public hospital-sponsored telemedicine platform: a case study in China. J Telemed Telecare. (2025) 31:265–76. doi: 10.1177/1357633X231176871 37309129

